# Supplementary material for: Peptidoglycan remodeling improves salt resilience of Zymomonas mobilis
Source: Appl Environ Microbiol. 2026 Mar 30;92(4):e02350-25. doi: 10.1128/aem.02350-25 (PMC13101528; doi:10.1128/aem.02350-25)
Supplement: Supplemental material — Fig. S1 to S6; Tables S1 to S4. [file aem.02350-25-s0001.docx]

**Supplemental figures**


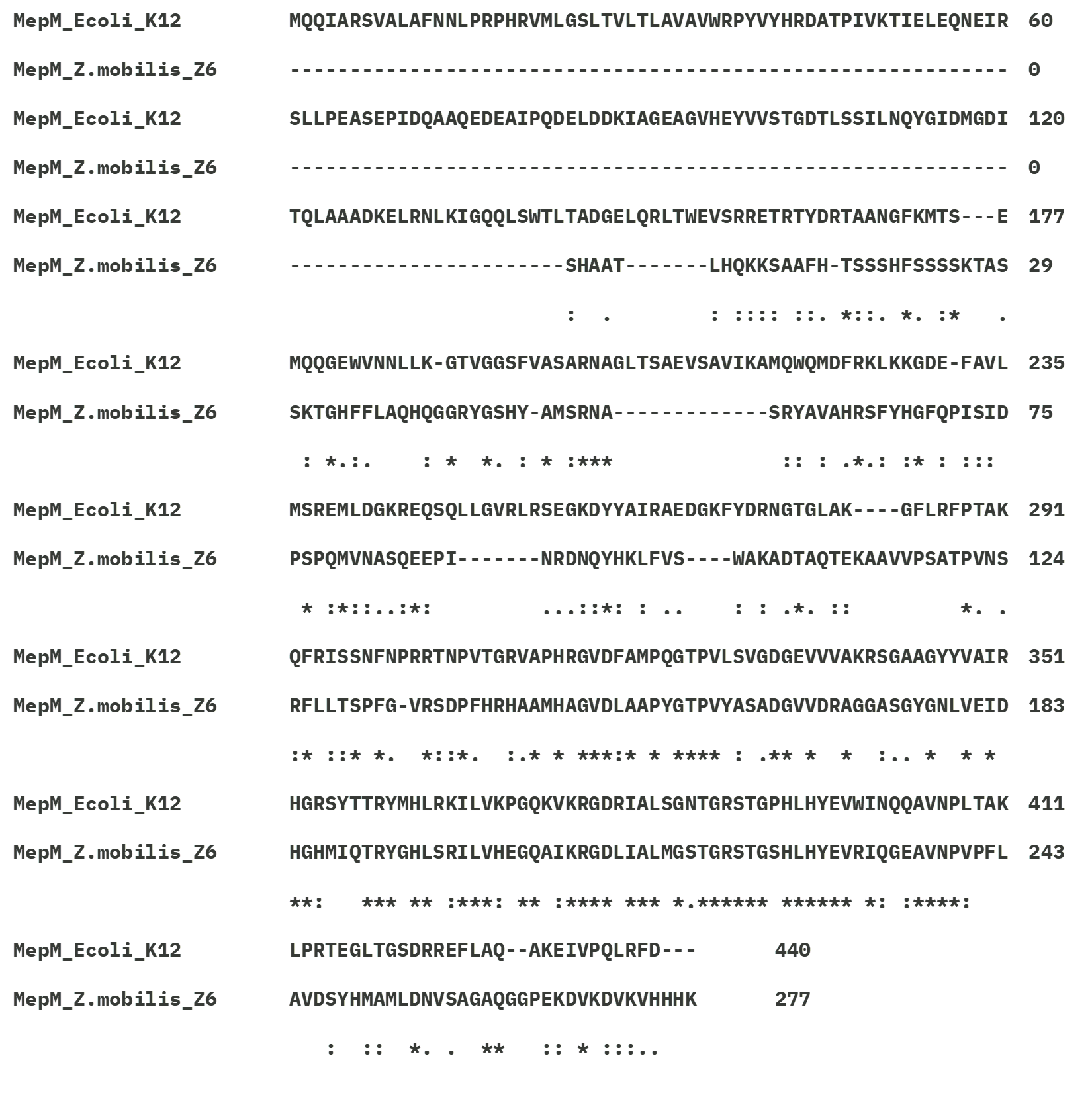


Fig. S1 Sequence alignment of *Z. mobilis* MepM with the sequences of *E. coli* K12 MepM, analysed by Pairwise Sequence Alignment (EMBOSS needle)


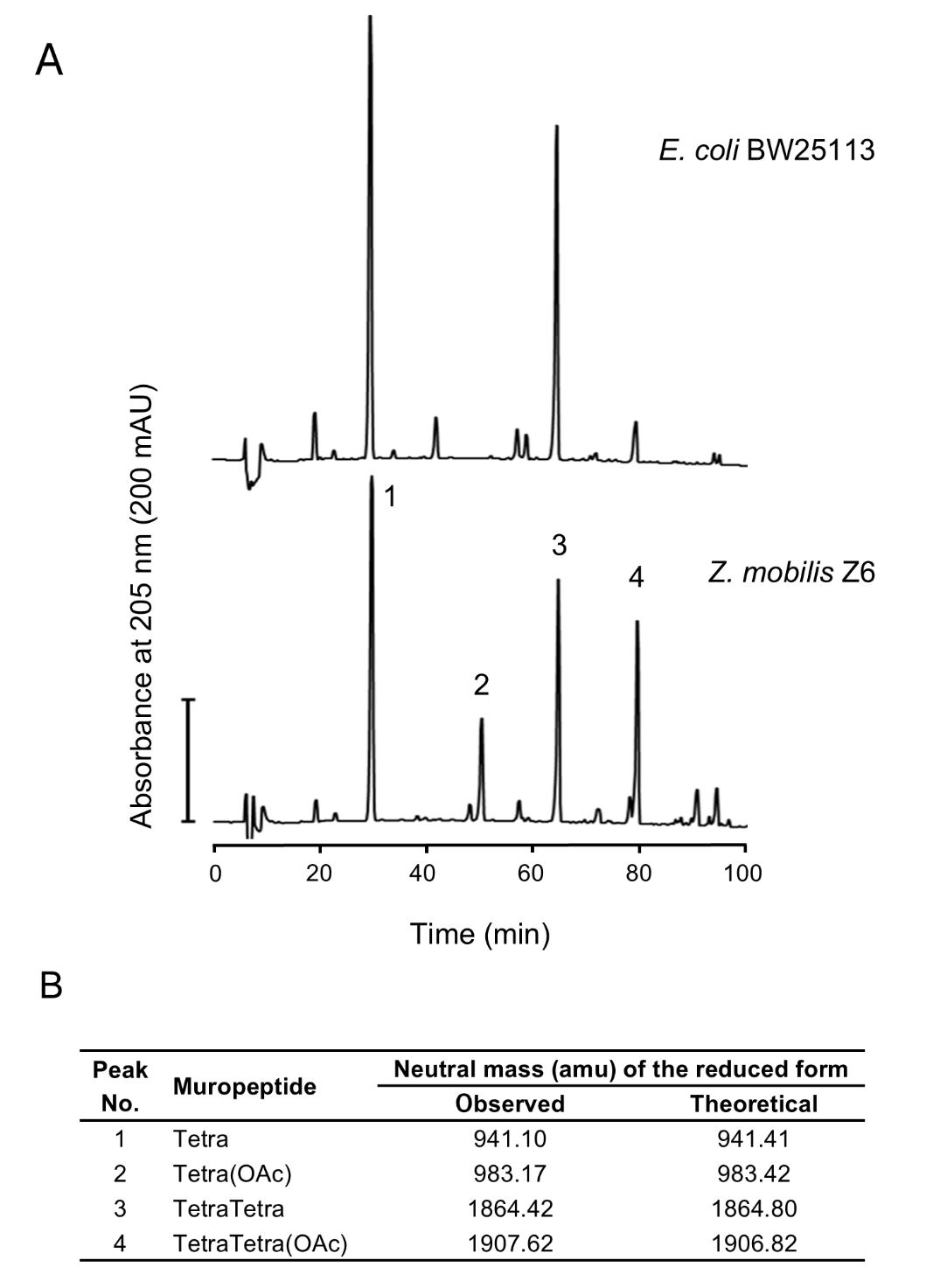


Fig. S2 Comparison of *E. coli* and *Z. mobilis* muropeptide profiles

(A) HPLC chromatogram of reduced muropeptides from *E. coli* BW25113 (top) cells and *Z. mobilis* Z6 cells (below). The used HPLC method is as described in (1). The labelled peaks are presented in the panel B. (B) Reduced muropeptides from *Z. mobilis* cells identified by Mass spectrometry. Theoretical masses were calculated using ChemDraw™ (PerkinElmerInformatics,UK)


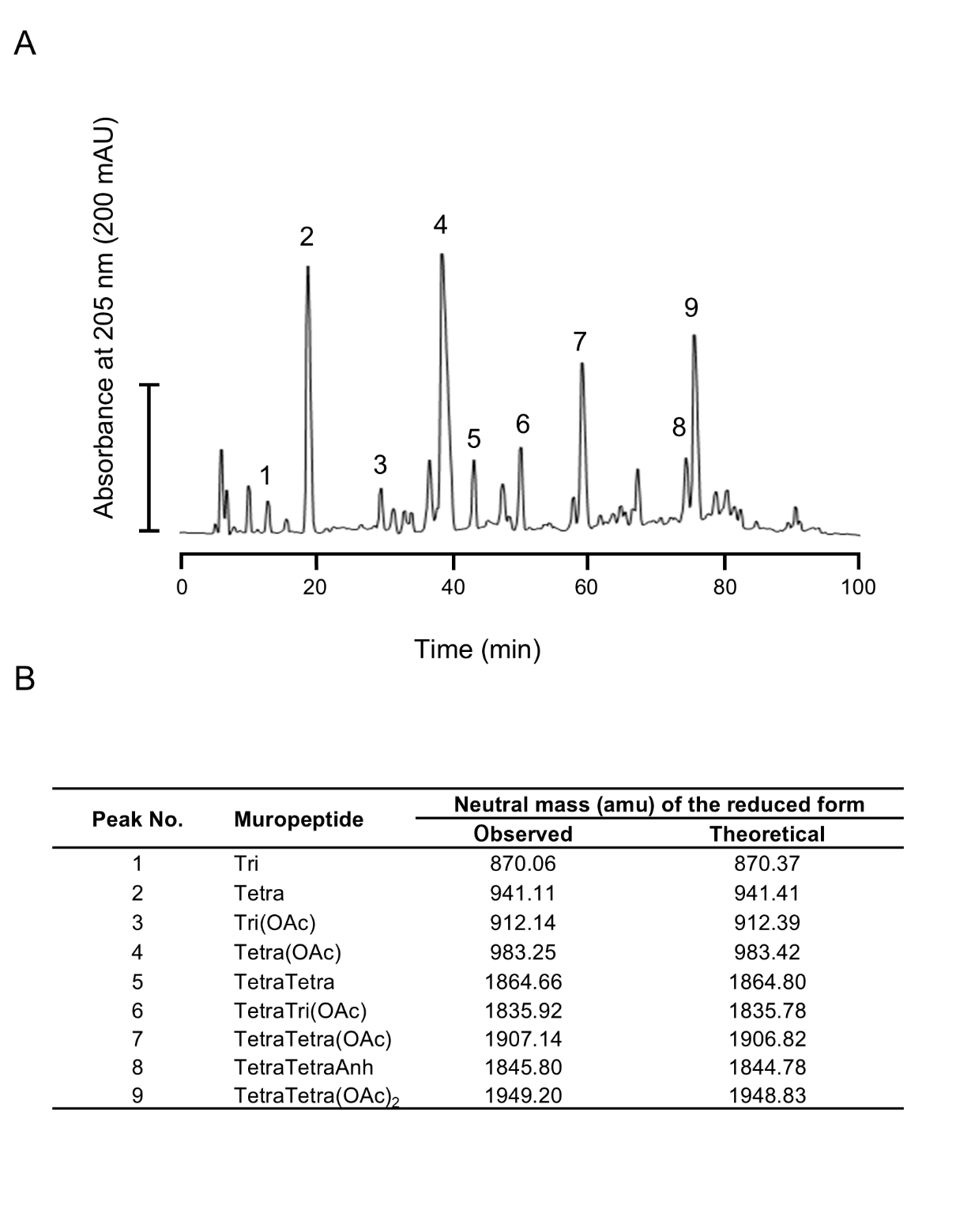


Fig. S3. Identification of *Z. mobilis* muropeptides

(A) HPLC chromatogram of reduced muropeptides from *Z. mobilis* Z6 cells. HPLC method was modified from (1) to preserve O-acetylated muropeptides. The labelled peaks are presented in the panel B. (B) Reduced muropeptides obtained from *Z. mobilis* cells. Theoretical masses were calculated using ChemDraw™ (PerkinElmerInformatics,UK). Muropeptides are numbered and their structures shown in Fig. S3


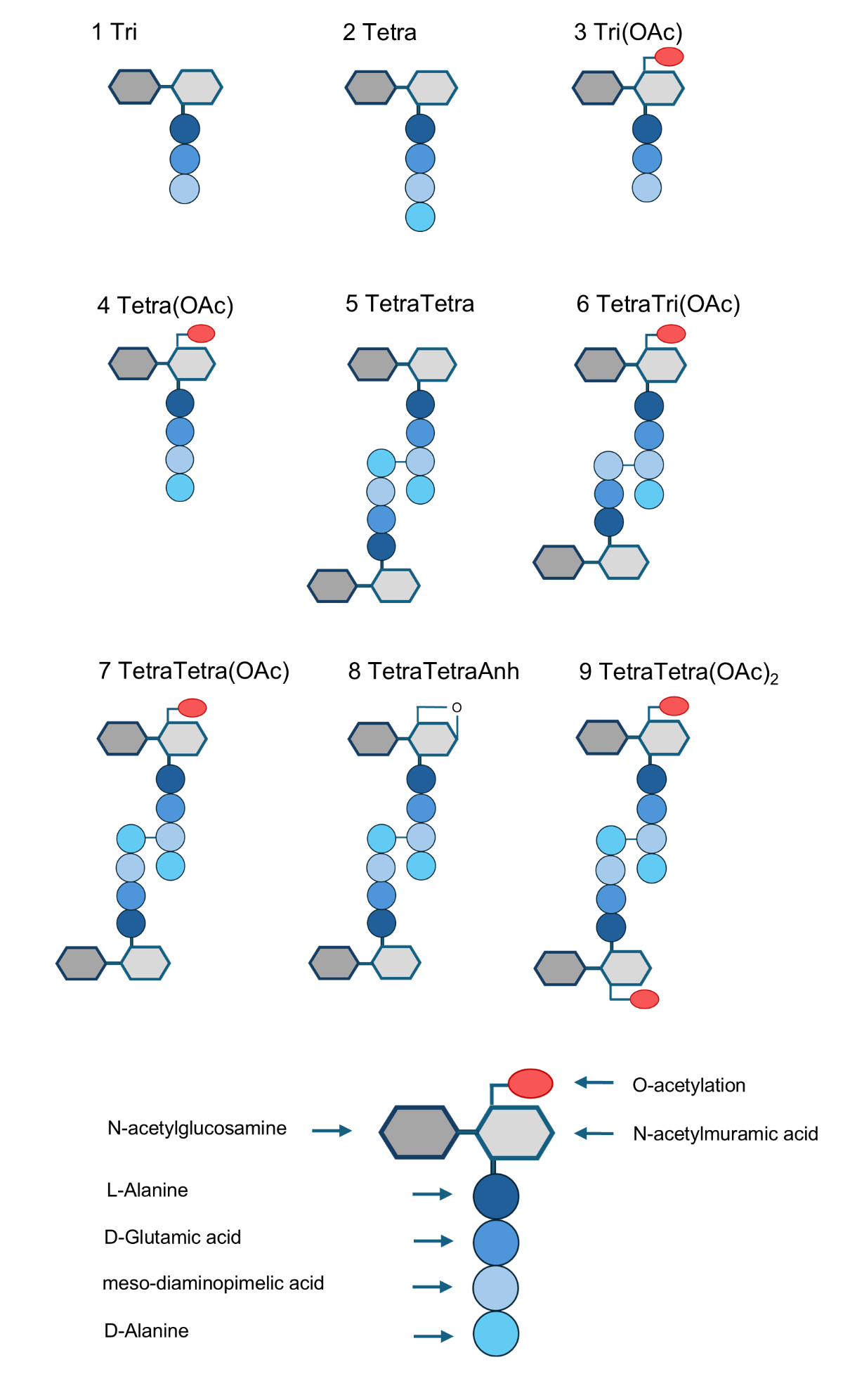


Fig. S4 Schematic structures of muropeptides released from *Z. mobilis* cells. The number corresponds to peak numbers in Fig. 4A, Fig. S3A, and Fig. S6.


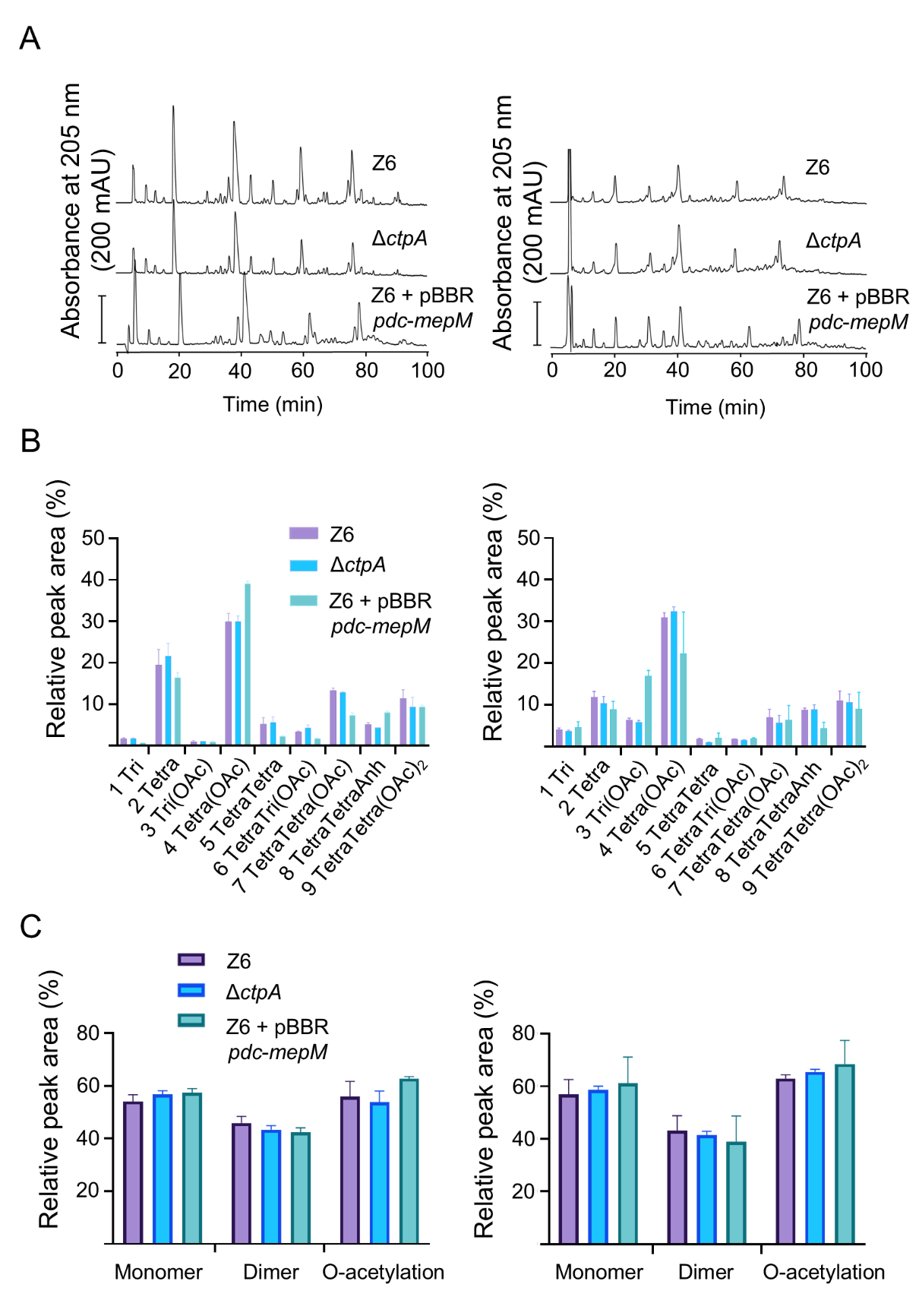


Fig. S5. Muropeptide profile of *Z. mobilis* strain Z6 (wild-type), Δ*ctpA* and Z6 *pdc*-*mepM*.

(A) Cells of Z6 (wild-type), Δ*ctpA* and Z6 *pdc*-*mepM* were grown under regular (left) and salt (right) conditions, and their PG were isolated. The muropeptides were released by cellosyl, reduced by sodium borohydride and separated by HPLC under O-acetyl group preserving conditions. The chromatograms of Z6 and Δ*ctpA* are duplicate from Fig 4A. (B) Relative quantification of muropeptides from Z6 and Δ*ctpA* grown under no-salt (left) and salt (right) conditions. (C) Relative proportion of monomeric, dimeric and O-acetylated muropeptides. Colours indicate the strains/growth condition.


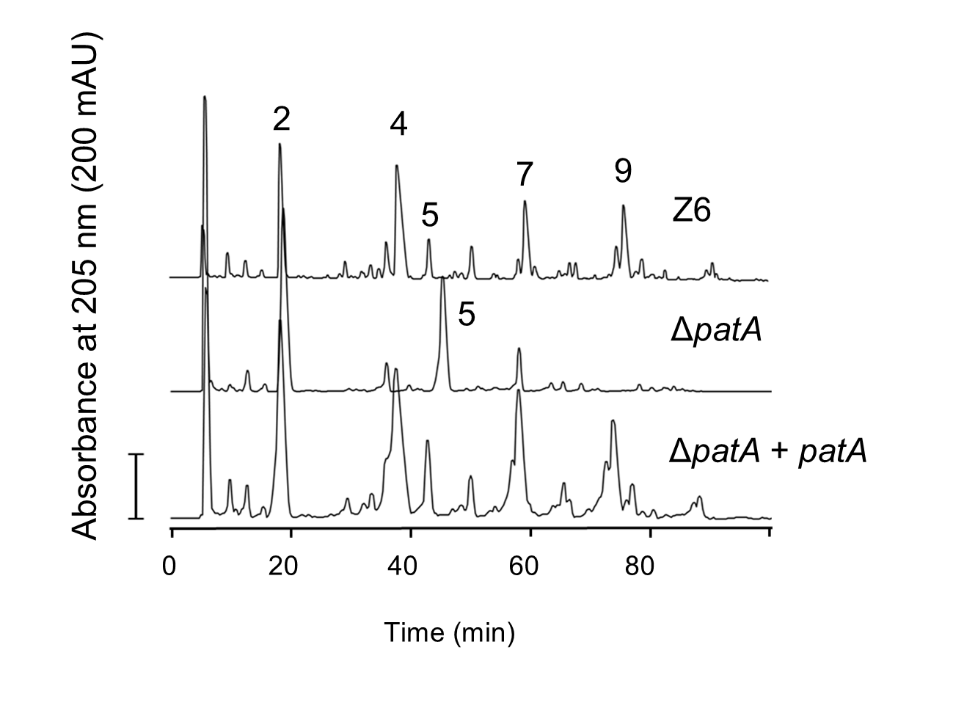


FIg.S6. Muropeptides profile of *Z. mobilis* strain Z6 (wild-type), Δ*patA* and Δ*patA* + *patA* (complemented strain). Cells of three strains were grown under regular growth conditions. The muropeptides from the cells were released by cellosyl, reduced by sodium borohydride and separated by HPLC under O-acetyl group preserving conditions. Be noted that the retention time of Peak 4 in Δ*patA* was shifted as this sample was run separately from the others. Muropeptides are numbered and their structures shown in Fig. S4.

**Supplementary tables**

Table S1. Bacterial strains used in this study.

| Bacterial strains |  |  | Genotype/ description/reference | | |
| --- | --- | --- | --- | --- | --- |
| *Zymomonas mobilis* Z6 | |  | ATCC29191 purchased from DSMZ | | |
| *Escherichia coli* Dh5a | |  | Cloning strain (Lab stock) | |  |
| *Escherichia coli* WM6026 | |  | Conjugation strain (2) | |  |
| *Zymomonas mobilis* Δ*ctpA* | |  | Δ*ctpA* (this study) |  |  |
| *Zymomonas mobilis* Δ*ctpA* + *ctpA* | | | Δ*ctpA* + *ctpA* (this study) | |  |
| *Zymomonas mobilis* Δ*patA* | |  | Δ*patA* (this study) |  |  |
| *Zymomonas mobilis* Δ*patA* + *patA* | | | Δ*patA* + *patA* (this study) | |  |
| *Zymomonas mobilis* pBBR | |  | Z6 + pBBR (this study) | |  |
| *Zymomonas mobilis* pBBR *pdc-mepM* | | | Z6 + pBBR *pdc*-*mepM* (this study) | | |
| *Zymomonas mobilis* pBBR *pdc-mepM* (H287A) | | | Z6 + pBBR *pdc*-*mepM* (H287A) (this study) | | |

Table S2. Plasmids used in this study.

| Plasmids |  |  | description/reference | |  |  |  |
| --- | --- | --- | --- | --- | --- | --- | --- |
| pPK15534 | |  | Suicide vector (2) |  |  |  |  |
| pPK15534 + *sacB* | |  | pPK15534 carrying *B. subtilis sacB* (this study) | | | |  |
| pPK15534 Δ*ctpA* | |  | pPK15534 carrying Δ*ctpA* cassettes (this study) | | | |  |
| pPK15534 + *ctpA* | |  | pPK15534 + *sacB* carrying *ctpA* insertion cassettes (this study) | | | | |
| pPK15534 Δ*patA* | |  | pPK15534 carrying Δ*patA* cassettes (this study) | | | |  |
| pPK15534 + *patA* | |  | pPK15534 + *sacB* carrying *patA* insertion cassettes (this study) | | | | |
| pBBR |  |  | Lab stock |  |  |  |  |
| pBBR *pdc*-*mepM* | |  | pBBR carrying *pdc* promoter and *mepM* (this study) | | | | |
| pBBR *pdc*-*mepM* (H287A) | | | pBBR *pdc*-*mepM* with the mutation H287A (this study) | | | | |

Table S3. Oligonucleotides used in this study.

| Oligo | Sequence |  |  |  | Use |
| --- | --- | --- | --- | --- | --- |
| **NKF1** | **TTTTTTCAACCACAAGATAAcgtatggggctgacttcaggtgc** | | |  | ***ctpA* mutation** |
| **NKF2** | **TGTTCAATGCCATCATGAATGTAATTCTCATGTTTGACAGCTTATCAC** | | | |  |
| **NKF3** | **cctgaagtcagccccatacgTTATCTTGTGGTTGAAAAAAATACCG** | | |  |  |
| **NKF4** | **ATTATCCCGAATTGTATTTTCAGTCTTTACTTTGTCCATCCATGACAG** | | | |  |
| **NKF5** | **GATGGACAAAGTAAAGACTGAAAATACAATTCGGGATAATAAGGATCGG** | | | |  |
| **NKF6** | **CTGTCAAACATGAGAATTACATTCATGATGGCATTGAACATTTCTTGGG** | | | |  |
| **NKF16** | **ATCGACCCGCTGGAATTGCCCcgtatggggctgacttcaggtgc** | | |  | ***patA* mutation** |
| **NKF17** | **TCCTAAAGCGGACATCGACAGTAATTCTCATGTTTGACAGCTTATCAC** | | | |  |
| **NKF18** | **cacctgaagtcagccccatacgGGGCAATTCCAGCGGGTCGATTTC** | | |  |  |
| **NKF19** | **TAGGCATAATGCCTTATTCCCCTATCTTGAAACCTAAATTTTACCCAAAAAAGGG** | | | |  |
| **NKF20** | **TTGGGTAAAATTTAGGTTTCAAGATAGGGGAATAAGGCATTATGCCTAATGAC** | | | |  |
| **NKF21** | **ATAAGCTGTCAAACATGAGAATTACTGTCGATGTCCGCTTTAGGACATTTATTTC** | | | |  |
| **NKF51** | **GTGATGGGTTAAAAAGGATCTTGGCGAAAATGAGACGTTGATCGGC** | | | | ***sacB* insertion into pKK15534** |
| **NKF52** | **AATTAACAGTTAACAAATAACCAGGGCTTCCCGGTATCAACAGGG** | | | |  |
| **NKF53** | **TCAACGTCTCATTTTCGCCAAGATCCTTTTTAACCCATCACATATACCTGC** | | | |  |
| **NKF54** | **TTGATACCGGGAAGCCCTGGTTATTTGTTAACTGTTAATTGTCCTTGTTC** | | | |  |
| **NKF99** | **acccgtggttcatgcatcagcgtatggggctgacttcaggtgc** | |  |  | **complementation *ctpA*** |
| **NKF100** | **ACGCCTTTTCTAGCAAAGGGGTAATTCTCATGTTTGACAGCTTATCAC** | | | |  |
| **NKF101** | **cctgaagtcagccccatacgctgatgcatgaaccacgggtgcag** | |  |  |  |
| **NKF102** | **CGCCCAAAAAATTGCAAGTCGTCGTTTTAGTTATATCTTGGGCTTGCTC** | | | |  |
| **NKF103** | **CAAGATATAACTAAAACGACGACTTGCAATTTTTTGGGCGAGCC** | | | |  |
| **NKF104** | **GGGGGTATAATCCGGTCTCATTATTCCCGACCCGATGGGGTGG** | | | |  |
| **NKF105** | **CCCCATCGGGTCGGGAATAATGAGACCGGATTATACCCCCTAGG** | | | |  |
| **NKF106** | **CTGTCAAACATGAGAATTACCCCTTTGCTAGAAAAGGCGTGCC** | | |  |  |
| **NKF219** | **cacctgaagtcagccccatacgctgatgcatgaaccacgggtg** | |  |  | ***patA* complementation** |
| **NKF220** | **AATCGCCCCCCATACGGGCAgtcgttttagttatatcttggcttgctctc** | | |  |  |
| **NKF221** | **gccaagatataactaaaacgacTGCCCGTATGGGGGGCGATTG** | | |  |  |
| **NKF222** | **gggggtataatccggtctcaATAACAATGCCCGTCCACAAGCCAG** | | |  |  |
| **NKF223** | **TTGTGGACGGGCATTGTTATtgagaccggattataccccctaggaac** | | |  |  |
| **NKF224** | **gctgtcaaacatgagaattacccctttgctagaaaaggcgtgcc** | |  |  |  |
| **NKF226** | **aatcatg TCTAGA TTCAAGGTGTCCCGTTCCTTTTTCCC** | | |  | ***pdc*-*mepM* in pBBR** |
| **NKF227** | **gtctttggcgatcatTGCTTACTCCATATATTCAAAACACTATGTCTG** | | |  |  |
| **NKF228** | **TATATGGAGTAAGCAatgatcgccaaagactctataaaaaatagagcg** | | |  |  |
| **NKF229** | **aatcatg CTCGAG ttatttatgatgatggactttgacatccttgac** | | |  |  |
| **NKF290** | **gcttacgaagtccgtattcagggcgaag** | |  |  | ***mepM* mutation H287A** |
| **NKF291** | **taaatggctacccgtcgaacgtcc** | |  |  |  |

Table S4. A list of enhanced proteins in Δ*ctpA* under salt conditions.

| Accession | Gene | gene annotation |  |  |  | log FC2 | P-value |
| --- | --- | --- | --- | --- | --- | --- | --- |
| AFN56367.1 | ZZ6_0468 | cell division protein FtsL |  |  |  | 1.61207698 | 0.00000005 |
| AFN57534.1 | ZZ6_1677 | endopeptidase MepM |  |  |  | 1.61082639 | 0.00000011 |
| AFN57370.1 | ZZ6_1505 | septum formation initiator DivIC | |  |  | 1.26758016 | 0.00000279 |
| AFN56779.1 | ZZ6_0886 | hypothetical protein transporter | |  |  | 1.18367341 | 0.00000608 |
| AFN56793.1 | ZZ6_0900 | ammonium transporter |  |  |  | 1.13329781 | 0.00003357 |
| AFN56550.1 | ZZ6_0653 | flagella basal body P-ring formation protein FlgA | | |  | 1.03044958 | 0.00035359 |
| AFN57348.1 | ZZ6_1483 | tonB-dependent siderophore receptor | | |  | 0.97573066 | 0.00353524 |
| AFN56826.1 | ZZ6_0933 | PepSY-associated TM helix domain protein | | |  | 0.94149198 | 0.00008456 |
| AFN57302.1 | ZZ6_1437 | extensin family protein |  |  |  | 0.90445173 | 0.00000368 |
| AFN57009.1 | ZZ6_1122 | methyl-accepting chemotaxis sensory transducer | | | | 0.90052160 | 0.00033675 |
| AFN56612.1 | ZZ6_0717 | signal transduction histidine kinase | |  |  | 0.89670457 | 0.00045371 |
| AFN57373.1 | ZZ6_1508 | phosphodiesterase I |  |  |  | 0.87069136 | 0.00006401 |
| AFN56081.1 | ZZ6_0177 | phosphatidate cytidylyltransferase | |  |  | 0.86008336 | 0.00299904 |
| AFN57484.1 | ZZ6_1625 | hypothetical protein |  |  |  | 0.84415919 | 0.00403771 |
| AFN56594.1 | ZZ6_0699 | succinate dehydrogenase membrane anchor subunit | | | | 0.83931356 | 0.00184612 |
| AFN57548.1 | ZZ6_1691 | gamma-glutamyltransferase | |  |  | 0.83708535 | 0.00000225 |
| AFN56142.1 | ZZ6_0238 | cellulose synthase catalytic subunit | |  |  | 0.83625218 | 0.00412790 |
| AFN56312.1 | ZZ6_0411 | protein of unknown function DUF192 | |  |  | 0.83507995 | 0.00104589 |
| AFN56649.1 | ZZ6_0754 | phosphoesterase PA-phosphatase | |  |  | 0.81428437 | 0.00074947 |
| AFN56237.1 | ZZ6_0336 | purine nucleoside permease | |  |  | 0.79774888 | 0.02641677 |
| AFN56944.1 | ZZ6_1056 | hypothetical protein |  |  |  | 0.79732337 | 0.00003339 |
| AFN57397.1 | ZZ6_1535 | TonB-dependent receptor | |  |  | 0.76353785 | 0.00033841 |
| AFN57255.1 | ZZ6_1390 | hypothetical protein |  |  |  | 0.75923528 | 0.00027323 |
| AFN56030.1 | ZZ6_0125 | potassium transport system protein Kup | | |  | 0.75355910 | 0.00000074 |
| AFN57022.1 | ZZ6_1136 | carbohydrate-selective porin OprB | |  |  | 0.75043935 | 0.00002096 |
| AFN56294.1 | ZZ6_0393 | capsular polysaccharide transport system permease | | | | 0.74682896 | 0.00000069 |
| AFN56660.1 | ZZ6_0765 | GtrA family protein |  |  |  | 0.72507108 | 0.00010170 |
| AFN56238.1 | ZZ6_0337 | Xanthine/uracil/vitamin C permease | |  |  | 0.72035548 | 0.00015460 |
| AFN56074.1 | ZZ6_0170 | CDP-diacylglycerol/serine O-phosphatidyltransferase | | | | 0.70684025 | 0.00011966 |
| AFN56585.1 | ZZ6_0690 | sodium:dicarboxylate symporter | |  |  | 0.69601679 | 0.00007877 |
| AFN56327.1 | ZZ6_0427 | hopene-associated glycosyltransferase HpnB | | |  | 0.69451356 | 0.03835694 |
| AFN56912.1 | ZZ6_1024 | TonB-dependent siderophore receptor | | |  | 0.66952645 | 0.00093194 |
| AFN56965.1 | ZZ6_1077 | S1/P1 nuclease |  |  |  | 0.65941698 | 0.00042556 |
| AFN57019.1 | ZZ6_1132 | major facilitator superfamily MFS_1 | |  |  | 0.64927937 | 0.01473404 |
| AFN57540.1 | ZZ6_1683 | hypothetical protein |  |  |  | 0.64912631 | 0.00210692 |
| AFN56863.1 | ZZ6_0973 | hypothetical protein |  |  |  | 0.64420395 | 0.00002604 |
| AFN56171.1 | ZZ6_0268 | phosphate ABC transporter | |  |  | 0.63982345 | 0.00006993 |
| AFN57344.1 | ZZ6_1479 | lytic transglycosylase MltA | |  |  | 0.63079272 | 0.00148274 |
| AFN56508.1 | ZZ6_0611 | ATP synthase subunit |  |  |  | 0.62623706 | 0.00102737 |
| AFN56329.1 | ZZ6_0429 | cation diffusion facilitator family transporter | | |  | 0.61363286 | 0.00000177 |
| AFN55993.1 | ZZ6_0088 | hypothetical protein |  |  |  | 0.61053082 | 0.02115910 |
| AFN56217.1 | ZZ6_0316 | peptidase S10 serine carboxypeptidase | | |  | 0.59359605 | 0.00014308 |

**References**

1. Glauner B. Separation and quantification of muropeptides with high-performance liquid chromatography. Analytical Biochemistry. 1988;172(2):451-64.

2. Lal PB, Wells FM, Lyu Y, Ghosh IN, Landick R, Kiley PJ. A Markerless Method for Genome Engineering in *Zymomonas mobilis* ZM4. Frontiers in Microbiology. 2019;Volume 10 - 2019.
